# Near-Field Sound Localization Based on the Small Profile Monaural Structure

**DOI:** 10.3390/s151128742

**Published:** 2015-11-13

**Authors:** Youngwoong Kim, Keonwook Kim

**Affiliations:** Division of Electronics & Electrical Engineering, Dongguk University-Seoul, Seoul 100-715, Korea; E-Mail: herokim@dongguk.edu

**Keywords:** sound localization, angle of arrival, monaural localization, acoustic resonance, cylindrical pipe, fundamental frequency, Cepstrum, single microphone, near-field

## Abstract

The acoustic wave around a sound source in the near-field area presents unconventional properties in the temporal, spectral, and spatial domains due to the propagation mechanism. This paper investigates a near-field sound localizer in a small profile structure with a single microphone. The asymmetric structure around the microphone provides a distinctive spectral variation that can be recognized by the dedicated algorithm for directional localization. The physical structure consists of ten pipes of different lengths in a vertical fashion and rectangular wings positioned between the pipes in radial directions. The sound from an individual direction travels through the nearest open pipe, which generates the particular fundamental frequency according to the acoustic resonance. The Cepstral parameter is modified to evaluate the fundamental frequency. Once the system estimates the fundamental frequency of the received signal, the length of arrival and angle of arrival (AoA) are derived by the designed model. From an azimuthal distance of 3–15 cm from the outer body of the pipes, the extensive acoustic experiments with a 3D-printed structure show that the direct and side directions deliver average hit rates of 89% and 73%, respectively. The closer positions to the system demonstrate higher accuracy, and the overall hit rate performance is 78% up to 15 cm away from the structure body.

## 1. Introduction

Conventional sound localization systems are focused on far-field source discrimination with multiple receivers. Plane wave radiation in the far-field area provides linear direction-wise information on the time and phase of signal propagations. Spatially distributed receivers collect the given cues for deterministic and/or stochastic processes in order to estimate the source direction. Numerous approaches have been addressed and realized in various domains over the far-field zone. The well-defined propagation models have devised high-performance localization systems in various mediums, such as sonar systems. In general, a higher number of receivers and further exploration of signals lead to improved estimation and detection results in localization.

The near-field condition is decided by the relative closeness to the sound source compared to the interest wavelength. The physics in the near-field region shows complicated travelling behavior because of the dominating non-propagating interference pattern from the immediate distance. In addition, the waveform distribution in the source proximity demonstrates non-plane propagation, which delivers degraded performance from typical localization algorithms. Far-field localization has been extended to the near-field condition using various methods. The near-field beamforming algorithms based on linear sensor array are addressed by the modification of subspace estimation approaches such as multiple signal classification (MUSIC) [1,2] and estimation of signal parameters via rotational invariance techniques (ESPRIT) [3,4]. The stochastic processes for beamforming are similarly employed for near-field localization as maximum-likelihood [5,6] and linear prediction [7]. Various configurations of the receiver array arrangement have been used for near-field localization, such as multiple subarrays [8], small aperture arrays [9], and heterogeneous sensors [10,11,12,13]. Moreover, the applications of near-field localization include, but are not limited to, speech processing [14,15,16] and sound source imaging [17].

Single microphone localization, known as monaural localization (ML), is based on the understanding of the pinna function. The localization system estimates the frequency modification delivered by the particular structure around the receiver similar to the pinna structure. Several studies have addressed the structure-enhanced localization system outlined below. An estimator of the arrival time between direct and indirect propagation was designed by analog circuitry for the pinna structure [18]. The pinna-like reflector and corresponding detection algorithm provided the extended dimension for the binaural system [19,20,21,22,23]. The directivity pattern of the head-related transfer function was improved by exploring the various structures around the microphone [24]. Non-structural ML actively utilized the characteristics of indoor speech propagation for situation-related localization [25,26,27]. The indoor condition exhibited position-dependent Cepstral and speech parameters, which could be further enhanced by the parabolic reflection structure for the ML system [28]. The audio-visual method based on Cepstral parameters was suggested by Friedland *et al*. for the hybrid ML system [29].

**Figure 1:**
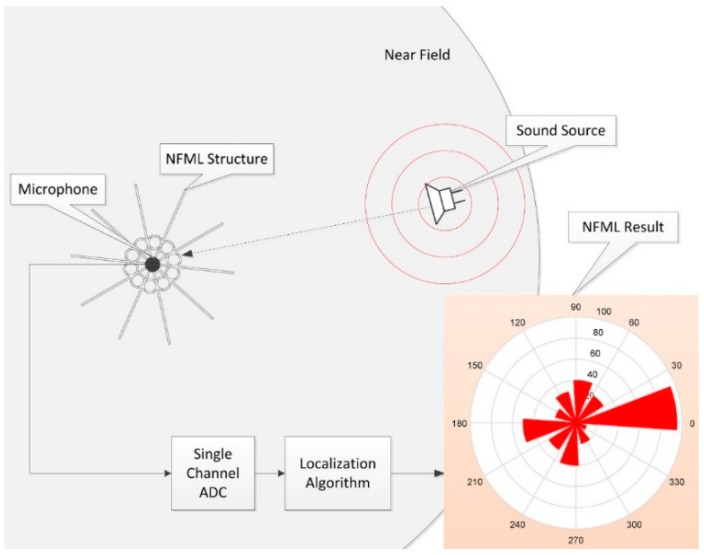
Functional diagram of NFML system.

This paper proposes a novel sound source localization system for a near-field situation with a single microphone. Near-field monaural localization (NFML) is made possible by using the dedicated asymmetric structure around the single receiver to induce the acoustic resonance. Instead of using the arrival times or phases, the resonance in an individual direction shows the particular fundamental frequency utilized to determine the direction in the proximity of the receiver. In a vertical fashion, ten pipes of different lengths envelop the single receiver over the cylindrical distribution to deliver the distinctive fundamental frequencies. The rectangular wings are positioned between the pipes in radial directions to prevent the excessive diffraction that creates localization blur. According to the near-field sound source and receiving open pipe, the particular fundamental frequency identifies the angle of arrival (AoA) based on the acoustic resonance. The Cepstral parameter is modified to evaluate the fundamental frequency. Once the system estimates the fundamental frequency of the received signal, the length of arrival and AoA are derived by the designed and evaluated model. The overall functional diagram is illustrated in Figure 1 with a single-channel analog-to-digital converter (ADC). The circles around the microphone represent the vertical pipes with different lengths that are propagated by the sound generated by a nearby source. The received signal is digitized by the ADC, and the results in terms of the likelihood of each direction are delivered by the discrete process of the localization algorithm. A higher value represents an elevated possibility of an AoA.

The cylindrical pipe profile is used for the fundamental structure of the NFML system, and the characteristic parameters of the structure are derived from the theory, simulations, and experiments. The initial design is obtained from acoustic resonance theory in order to determine the individual pipe size and distribution. The simulation result by COMSOL Multiphysics software analyzes the acoustic effect of the multiple pipe arrangement on the NFML structure. The further acoustic experiments in the anechoic chamber enable the adjustment of the overall structure and the verification of the simulations and theory. Several design iterations of the procedure and mutual feedback from the theory to the experiments render the structure reliable in terms of performance.

The aim of this paper is to extend the authors’ previous work on the ML for far-field sound sources based on pyramidal horns [30]. The horizontal arrangement of the horns denoted the relatively decoupled acoustic interference between inlets for enhanced performance; however, the ML profile reduced the maneuverability due to the oversized structure. For near-field sound sources, the NFML improves the overall size and accuracy with extensive simulations and experiments on the structure. Other works on the subject by the authors are also related and expanded for progress in localization, such as azimuthal movement detection based on a binaural architecture [31] and target localization algorithm over distributed acoustic sensor network [32]. The earlier works concentrated on temporal processing for localization, and this paper focuses on further spectral processing from the reduced propagation structure. In addition to the acoustic model simulation, experiments are performed and evaluated within the same anechoic chamber [33] used in the previous works.

## 2. Methodology

A cylindrical pipe creates a length-wise frequency response known as the fundamental frequency, which is the frequency periodicity in every fundamental frequency spectrum distance. The fundamental frequency is inversely proportional to the pipe length, and the well-known equation for the fundamental frequency of an open-ended cylindrical pipe is presented below, where *c* is the sound speed and *L* is the pipe’s longitudinal length. This paper employs a sound speed of 346 m/s, which is the conventional speed at an air temperature of 25 °C.

(1) $$f_{fund}\left(L\right)=\frac{c}{2L}$$

**Figure 2:**
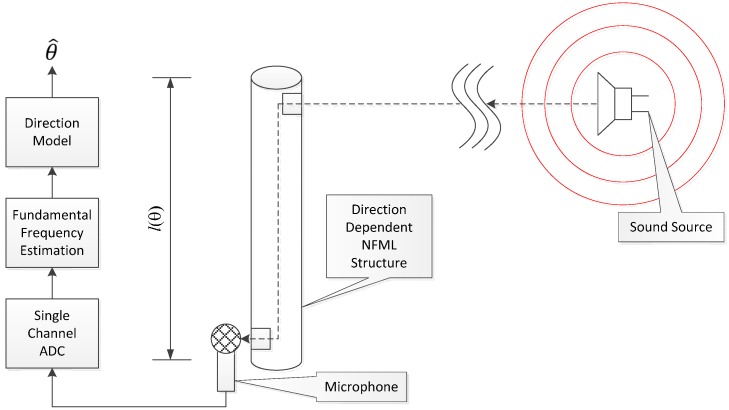
System architecture of proposed NFML system.

Once the structure of the pipe is established, the fundamental frequency can be derived by the above equation and *vice versa*. The NFML structure adopts multiple cylindrical pipes with a range of longitudinal lengths to produce the perceptible fundamental frequencies. The pipes are organized in a vertical manner to maintain a manageable profile and placed around the microphone to keep the equidistance between the pipe outlets and microphone receiver. The signal that properly propagates a certain pipe generates the unique fundamental frequency that is estimated by the dedicated algorithm in the NFML system. The localization decision is made by the predetermined direction model between the fundamental frequency and the corresponding direction based on the structure configuration. The overall procedure of the NFML system is depicted in Figure 2 below.

The fundamental frequency estimation is performed by the Cepstral parameter previously used by the authors [30]. The cycle frequency between the peak responses in the frequency domain is computed by a double discrete Fourier transform (DFT) as Cepstrum. For a received and digitized sequence *x*[*n*], Equation (2) represents the DFT with data length *N* to transfer the domain toward the frequency. In Equation (3), the second transform executes the DFT over the logarithm magnitude of the frequency information to obtain the spectrum periodicity. The second transform result delivers the fundamental frequency found by Equation (4). A higher $\hat{X}\left[r\right]$ magnitude presents a stronger periodicity in the frequency distribution of the *r* value; hence, by applying the maximum argument operation, the fundamental frequency is estimated in Equation (4). The range of *r* values can be limited for possible fundamental frequency searching within the potential candidates given by the pipe configuration; however, the minimum and maximum *r* values are 1 and *N*/4, respectively. The overall procedure for the Cepstral parameter involves the equations below.

(2) $$X\left[k\right]=\;\overset{N-1}{\underset{n=0}{\sum }}x\left[n\right]e^{-j\frac{2\pi }{N}kn}$$

(3) $$\hat{X}\left[r\right]=\overset{\frac{N}{2}-1}{\underset{k=0}{\sum }}\log_{2}\left|X\left[k\right]\right|e^{-j\frac{2\pi }{\left(\frac{N}{2}\right)}rk}$$

(4) $$r_{fund}=\underset{r=\left\{r\in \mathbb{Z}|r_{\min}\leq r\leq r_{\max}\right\}}{\arg\max}\left|\hat{X}\left[r\right]\right|$$

The discrete processing of the Cepstrum operates the information based on the integer sequence. Therefore, a proper interpretation is required to obtain the physical unit from the index, and it is expressed as:

(5) $${\tilde{f}}_{fund}=\frac{f_{s}}{2r_{fund}}$$

where *f_s_* is the sampling frequency of the given *x*[*n*] sequence and ${\tilde{f}}_{fund}$ is the estimated fundamental frequency. The *r* values of the second DFT from Equation (3) indicate the scaled time, and an inverse operation is required to obtain the fundamental frequency in Equation (5). Further detail on the derivation can be found in Appendix A of a previous paper [30].

**Figure 3:**
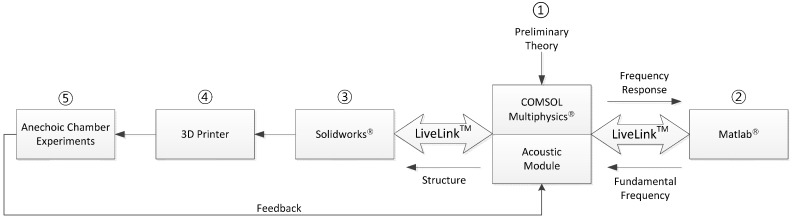
Workflow of NFML system development.

The structure of the NFML is the combination of multiple apertures to produce the distinct frequency response from an individual direction signal. The mutual interference between the substructures yields the complicated architectural acoustics that may lead to the unexpected spectral pattern and estimation performance degradation. Furthermore, the absence of a dominant equation for the comprehensive NFML structure is the design problem in the workflow. This paper extensively utilizes the procedure of modeling, simulation, and experiment to reduce design mistakes and experiment iterations. Figure 3 displays the overall procedure to design and evaluate the NFML structure.

For easy information access, the COMSOL Multiphysics simulator supports the direct connection to various programs, such as Matlab, Solidworks, *etc*. The designed preliminary structure based on Equation (1) is assessed by the simulator to compute the frequency response. Matlab provides parametric variations and receives the frequency response to calculate the fundamental frequency from the Cepstral parameter. For the target structure, the range of parameter values can be executed on the simulator and program simultaneously as batch processing for optimal value searching. Procedures ① and ② in Figure 3 represent the simulation workflow for comprehensive structure design. The candidate structure from the simulation is realized by the 3D design program and printer for acoustic experiments in the anechoic chamber. The Solidworks program seamlessly retrieves the 3D design from COMSOL and provides the cosmetic modification for a 3D-printable shape. The printed shape is placed in the anechoic chamber to analyze the received spectrum and examine the localization performance. Procedures ①, ③, ④, and ⑤ in Figure 3 represent the experiment workflow for the comprehensive structure design. Based on the acoustic performance, further adjustments might be required for several iterations.

## 3. NFML Structure Design and Simulation

This section presents the design procedure of the NFML structure from an individual pipe to a complete configuration. The theoretical background begins with a coarse outline, and the simulation refines the architecture for optimal performance in fundamental frequency and localization accuracy. The designed structure in this section serves as the final shape for the NFML system with potential further adjustment based on the direction model presented in Section 4. Note that the NFML structure indicates the physical structure, and the NFML algorithm denotes the spectral estimation algorithm with the direction model. In addition, the NFML system is the combination of the structure and the algorithm.

### 3.1. Individual Cylindrical Pipe

**Figure 4:**
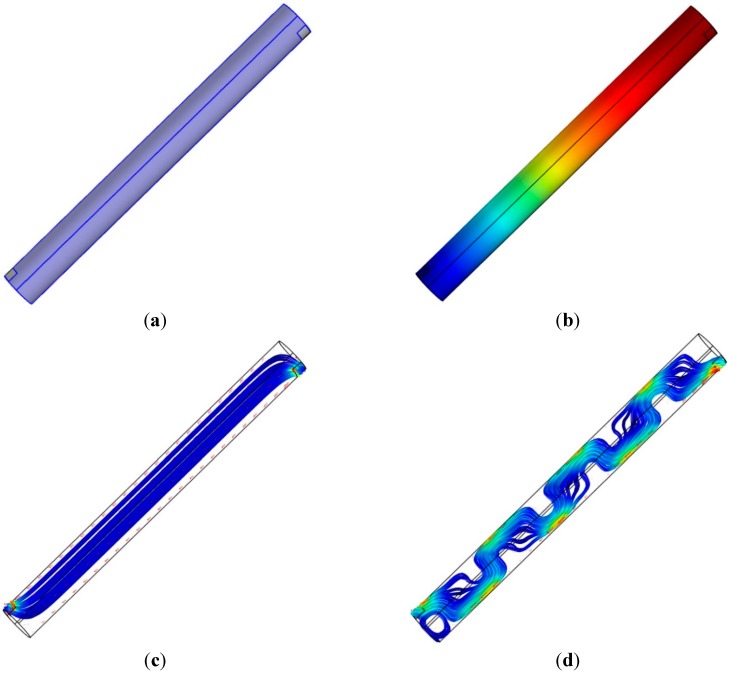
Simulation with single cylindrical pipe structure: (**a**) physical structure (pipe dimension: *l* = 117.7 mm and *d* = 10 mm; window dimension: *h* = 3 mm and *w* = 8 mm); (**b**) sound pressure distribution at 1033.6 Hz; (**c**) root mean square (RMS) sound intensity streamline at 1033.6 Hz; and (**d**) RMS sound intensity streamline at 22,007 Hz.

The acoustic resonance from the cylindrical pipe considers the open-end model, in which both ends of the pipe are fully open. The vertical configuration of the pipe requires the side inlet and outlet of the signal to create the resonance. For a pipe of a given length, one top side of the longitudinal face includes the rectangular signal inlet, and the other bottom side of the face has the outlet for signal reception by the NFML microphone. Both ends of the pipe are completely closed, as shown in Figure 4a. The simulation is accomplished in the condition that the plane wave is introduced from the inlet and the propagated plane wave is freely leaving through the outlet. The processing length for the simulation and experiment is 1024 samples as data window size. The simulator produces the outcome in every spectral resolution as the sampling frequency divided by the data window size (44,100 Hz/1024) up to half of the sampling frequency.

For the 1033.6 Hz of the simulation upshot, the sound pressure distribution is represented by the color-coded plot in Figure 4b, and the wavelength (334.8 mm) of the given frequency suitably corresponds to the distribution. The sound intensity streamline from Figure 4c illustrates the proper propagation of the given frequency wave from and to the open windows. The longitudinal motion of the propagation and open windows of the pipe present the predictable length-wise fundamental frequency, as shown in Equation (1) in the designed structure.

Figure 4d demonstrates the complicated propagation pattern in the sound intensity streamline for the intended frequency at 22,007 Hz. The acoustic wave, which has a smaller wavelength (15.7 mm) than twice the pipe diameter (2 × 10 mm), provides the higher-order waves in non-longitudinal directions. For the low-frequency waves, the longitudinal motion is dominant along the principal axis, as shown in Figure 4c; however, the transverse modes start to be excited in the high-frequency waves due to the higher-order waves along all directions. Figure 4d presents the situation of effectively longer travel paths that cause the lower fundamental frequency. According to the multi-dimensional propagation, the acoustic response of the pipe is assumed to generate a fluctuating and complicating pattern in the high-frequency region.

**Figure 5:**
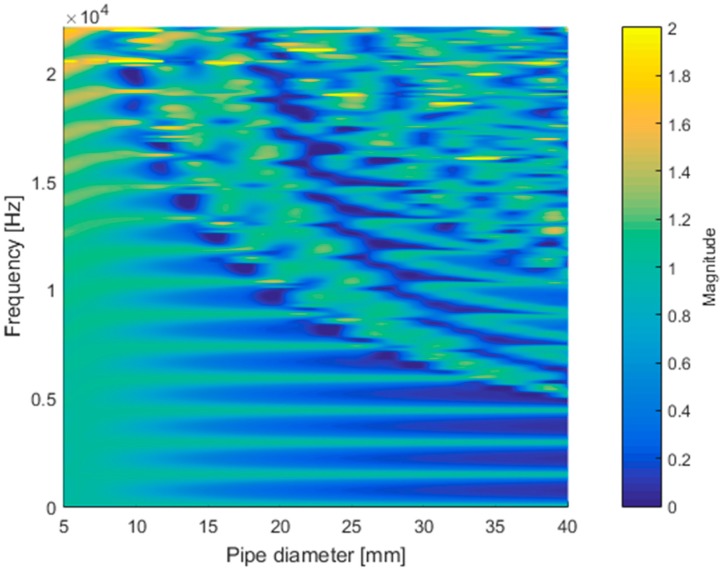
Simulated frequency response of cylindrical pipe (*l* = 117.7 mm, *h* = 3 mm, and *w* = 8 mm) with various diameters.

A further simulation for the pipe diameter of the given structure is performed and illustrated in Figure 5. The simulation increased the diameter without changing any other parameters of the structure and provided the corresponding frequency response up to the half of the sampling frequency. The frequency response is defined as the ratio between the outgoing and incoming acoustic energy that denote the outgoing power at the outlet and the incoming power at the inlet, respectively. The higher value in the frequency response responds to the amplified output for the selected frequency. The parametric frequency study indicates the prevailing non-longitudinal propagation for increased diameter and high frequency as the irregular pattern in the upper-right-hand corner of Figure 5. In the small diameter pipe, the frequency response shows a shallow but regular outline in terms of fundamental frequency over the whole range of the frequency. At around 10 mm, the regularity of the fundamental frequency starts to collapse from the high-frequency zone. However, the prominence of the regularity contrast is improved further for the larger-diameter pipes.

**Figure 6:**
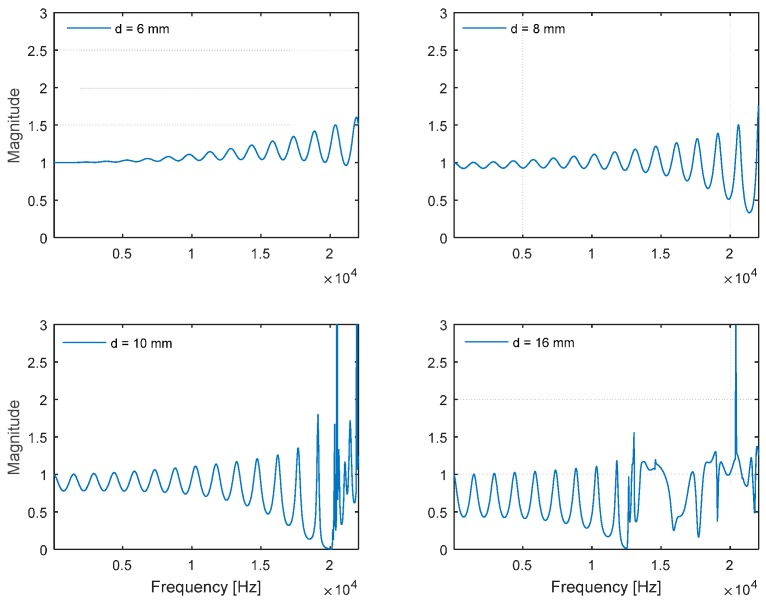
Plot matrix for simulated frequency response of cylindrical pipe (*l* = 117.7 mm, *h* = 3 mm, and *w* = 8 mm) with selected diameters.

The individual plot matrix of the frequency responses for the given diameters is depicted in Figure 6. Up to a diameter of 8 mm, the frequency response delivers the periodic sinusoidal wave over the entire frequency range. The response for a diameter of 10 mm includes rapid fluctuation above the 20 kHz area, and the phenomenon becomes worse for the larger-diameter pipe, as shown in the 16-mm plot. The second DFT in the Cepstrum is the additional Fourier transform to analyze the periodic component in the frequency information from the first DFT. The pure and noticeable sinusoidal form of the signal anticipates the generation of the prominent fundamental frequency distribution in the estimation process. The 6-mm and 8-mm frequency responses are relatively uncontaminated, but the signals indicate weak amplitudes overall. As the signal becomes stronger by increasing the diameter, the complex pattern in the response is also expected to enlarge the area, which causes the multiple side-lobes to blur the estimation result. A tradeoff is required to balance the purity and magnitude for an optimal estimation outcome.

**Figure 7:**
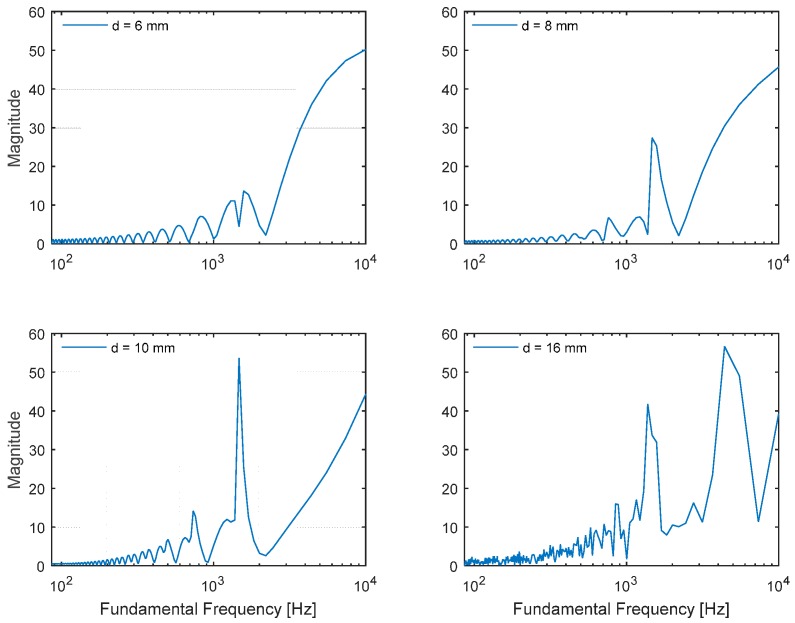
Plot matrix for simulated fundamental frequency of cylindrical pipe (*l* = 117.7 mm, *h* = 3 mm, and *w* = 8 mm) with selected diameters.

Instead of processing entire frequencies with Equation (3), the range-limited DFT is employed to compute the fundamental frequency in the Cepstral parameter. The frequency span is limited to up to 20 kHz, which is an acceptable standard range of audible frequency used for conventional audio equipment. The Cepstral parameter with a limited range is applied to the selected diameter pipes in Figure 7. The weak magnitude of the frequency response from the narrow pipes demonstrates the low contrast on the fundamental frequency plot. On the contrary, the contaminated magnitude from the wide pipes shows the redundant strong side-lobes to mislead the fundamental frequency estimation. The 10-mm-diameter pipe delivers the optimal performance to estimate fundamental frequency according to the simulation.

**Figure 8:**
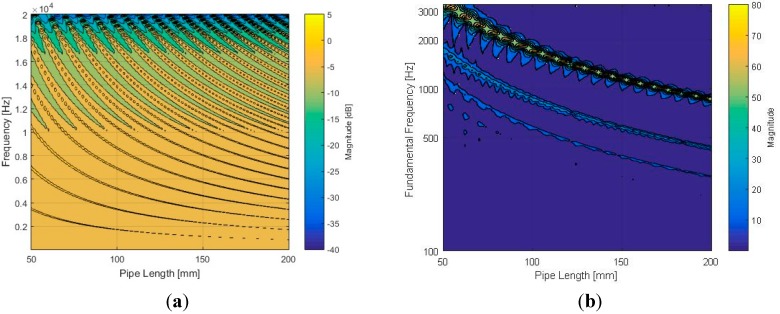
Simulated results for cylindrical pipe (*d* = 10 mm, *h* = 3 mm, and *w* = 8 mm) with various lengths: (**a**) frequency response in decibel scale and (**b**) fundamental frequency distribution in absolute scale.

Figure 8 displays the frequency response and fundamental frequency plot for a range of pipe lengths with a 10-mm diameter. Note that both plots use the frequency up to 20 kHz. In addition, the magnitudes for the frequency response and fundamental frequency use the decibel and absolute scale, respectively. Figure 8a demonstrates the existence of spectrum periodicity that becomes dense and cyclical populations for long-length pipes. Consequently, the Cepstral parameter for the identical structure configuration represents the decreasing fundamental frequency moving toward the right of Figure 8b. The one-half and one-third values of the fundamental frequency are visualized with blurry lines in addition to the dominant fundamental frequency threads, because they are also the weak fundamental frequencies that respond to certain periodicities of the signal.

**Figure 9:**
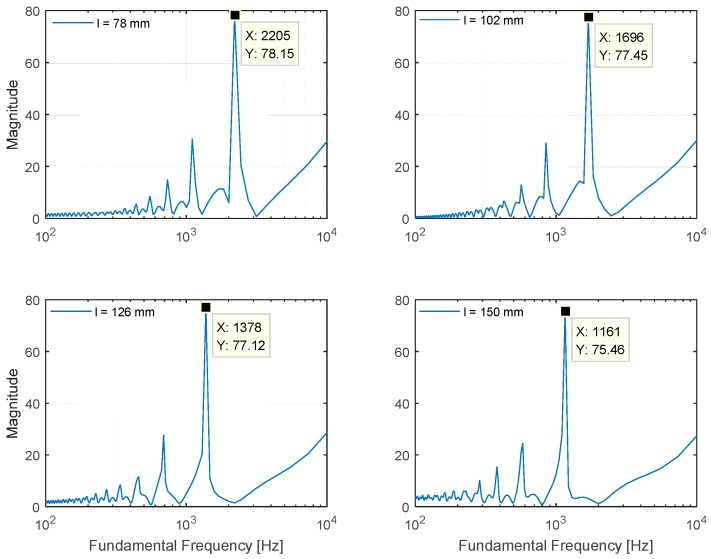
Plot matrix for simulated fundamental frequency of cylindrical pipe (*d* = 10 mm, *h* = 3 mm, and *w* = 8 mm) with selected lengths.

The fundamental frequency plots of the selected lengths are organized in Figure 9. According to the figure, the estimated fundamental frequency appropriately satisfies the relation defined by Equation (1). The difference between the computed and simulated value originates from the discrete spectral resolution of the Cepstral parameter modification given by Equation (5). For example, the length of 78 mm provides the 2218 Hz (346,000/(2 × 78)) fundamental frequency according to Equation (1), and the value is sampled by the nearest frequency 2205 Hz (44,100/(2 × *r*); *r* = 10) from Equation (5). Therefore, the simulation result complies well with the theoretical model that creates the distinct fundamental frequency by modifying the cylindrical pipe length. The designed cylindrical pipe with inlet and outlet windows fulfills the primitive architecture of the NFML structure, and it is modified further for azimuthal localization in the next subsection.

### 3.2. NFML Structure

This subsection discusses and presents the entire structure of the NFML based on the cylindrical pipe from the previous subsection. The vertical arrangement of the cylindrical pipes is used to build the NFML structure for the low profile. The large-dimension cylindrical pipe in terms of length and diameter sustains the multi-directional pipes with individual lengths for fundamental frequency variation. The inlet window of the directional pipes faces outside toward the independent radial path to receive the acoustic signal from the dedicated direction. The outlet window of the directional pipe faces inside the large pipe to collect the signal for a single microphone. The center-located pipe has a sufficiently long length for a low fundamental frequency to avoid estimation collision and a diameter that provides enough horizontal space to arrange multiple pipes around the center body. With the given outline; the NFML structure is expected to provide the direction-wise fundamental frequency to localize the AoA properly.

**Figure 10:**
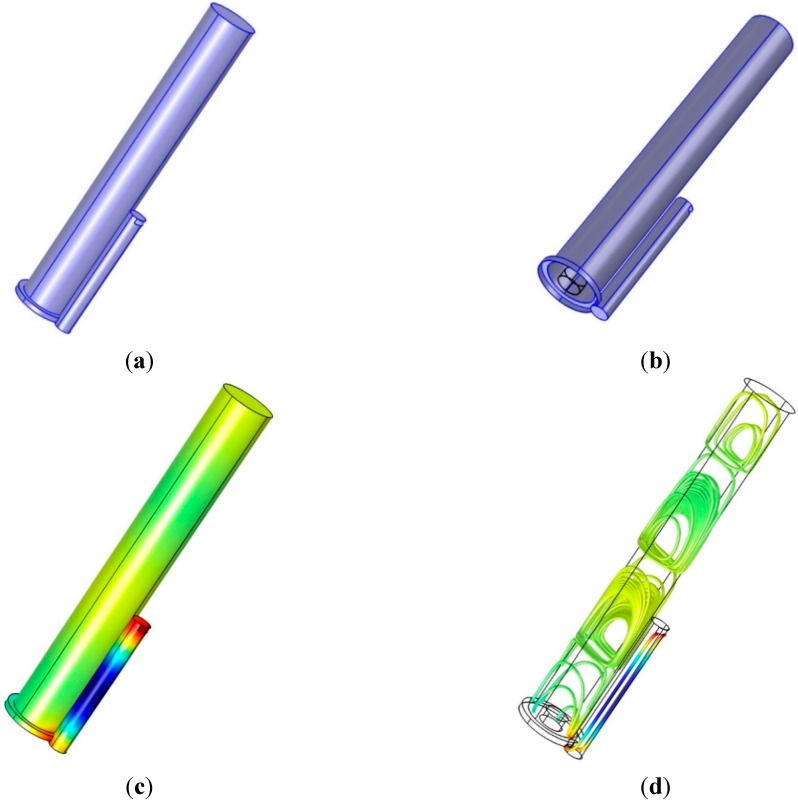
Simulation with NFML structure with single directional pipe: (**a**) physical structure (center pipe dimension: *l* = 192.1 mm and *d* = 27.8 mm; directional pipe dimension: *l* = 78.4 mm and *d* = 10 mm); (**b**) at the bottom of the NFML structure, the transparent cylinder indicates the microphone; (**c**) sound pressure distribution at 3014.6 Hz and (**d**) RMS sound intensity streamline at 3014.6 Hz.

The directional pipe with a cylindrical structure was analyzed to produce the predictable fundamental frequency in the previous subsection. The further simulation with the suggested structure in this subsection presents the numerical decision for directional pipe lengths. The dimensions of the center pipe are determined to initiate the overall NFML structure. The diameter of the center pipe is 27.8 mm for attaching the ten directional pipes around the body, and the length is 192.1 mm for responding to the 900 Hz fundamental frequency that is sufficiently far from the directional pipe producing the frequencies. The 900 Hz fundamental frequency moves to the nearest value 918.8 Hz (44,100/(2 × *r*); *r* = 24) by Equation (5) in the estimation process. Figure 10a illustrates the center pipe with one directional pipe that contains the inlet at the upper-right-hand side of the pipe and the outlet at lower-left-hand side of the pipe. The outlet window is not explicit in the figure, since the window directly connects to the center body. Figure 10b depicts the location of the microphone, which is the transparent cylinder at the bottom of the center pipe to collect the consistent signals from the directional pipes.

The simulation in Figure 10 is executed with a 78.4-mm-long pipe on the side to deliver primitive insights into the NFML structure. In Figure 10c, the sound pressure distribution is excited by a 3014.6 Hz signal. The strong and weak red colors on the windows indicate the signal inflow and outflow, respectively. According to the wavelength of the given frequency, the distribution is suitable for the 78.4-mm directional pipe; however, the acoustic movement on the center pipe is implicit with relatively flat color spreading. Figure 10d visualizes the sound intensity streamline at the identical frequency, and the periodic motions in the center pipe are expected to create a lower fundamental frequency than the directional pipe value.

**Figure 11:**
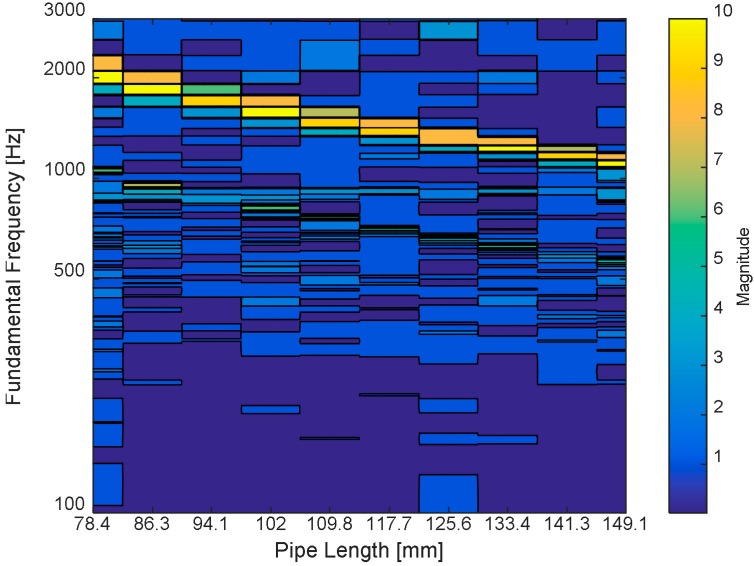
Simulated fundamental frequency distribution of NFML structure for various lengths.

According to the limited estimation resolution in the fundamental frequency by Equation (5), the range of *r* values is selected from 10 to 19, which corresponds to the fundamental frequency denoted by the second row of Table 1. The consequent lengths of the directional pipes are computed by Equation (1) and demonstrated in the *x*-axis of Figure 11 and third row of Table 1. Note that the adjacent pipe length difference is approximately 7.85 mm derived from the *c*/*f_s_* that is the combination of the two relationships between Equation (1) and Equation (5). The fundamental frequency resolution of Equation (5) and the length difference of 7.85 mm are consistent unless the sampling frequency *f_s_* or sound speed *c* is changed. The window size for the Cepstral parameter does not alter the resolution or difference either.

The simulation outcome for the designated directional pipes in the NFML structure is shown in Figure 11. The results are calculated and gathered by the single model as shown in Figure 10 that includes the center pipe with one directional pipe, whose length is changed to represent the corresponding fundamental frequency. As the pipe length is increased, the fundamental frequency gradually decreases to indicate the individual variation for the estimation process. The perceptible differences in the fundamental frequency distribution are observed in Figure 11. Moreover, note that the one-half value of the fundamental frequency is faintly illustrated below the primary line, and the fundamental frequency of the center body 918.8 Hz is indicated by the low contrast line beneath 1000 Hz. For the given range, the fundamental frequencies from the directional pipes and from the center body do not provide any overlap that could possibly generate confusion in the estimation procedure. Therefore, the simulation result validates the NFML structure for acoustic localization by fundamental frequency.

**Figure 12:**
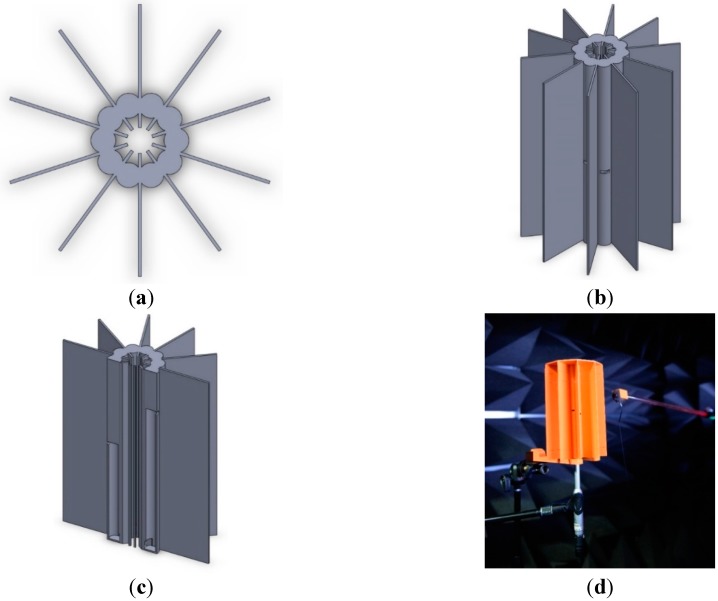
Designed NFML structure: (**a**) top view; (**b**) side view; (**c**) cross-sectional view; and (**d**) acoustic experiment in anechoic chamber with 3D-printed NFML structure.

Figure 12 presents the NFML structure with devised lengths for the pipes and center body. The proposed NFML structure in the figure includes the rectangular wings between the individual pipes to avoid significant acoustic diffractions. The small profile cylindrical structure is naturally vulnerable to acoustic diffraction, and it easily propagates the broadband signal to the other side with less attenuation [34]. The diffraction supplies a considerable amount of signals in the non-line-of-sight (NLoS) directions; hence, the estimation is substantially contaminated in the localization procedure. The radial length of the wing is 5 cm from the joint of two pipes, and the longitudinal length reaches both ends of the pipes, as shown in Figure 12b. Around a 192.1-mm-long pipe, ten directional pipes are located vertically to receive and collect the signal to the microphone. Each directional pipe has a particular length for the fundamental frequency, as illustrated in Figure 12c. The designated length is represented by the hollow area in the pipe, and the rest of the length is occupied by the material for symmetric structure to obtain the consistent response except fundamental frequency.

The NFML structure is realized by a 3D printer (Replicator 2, MakerBot, Brooklyn, NY, USA) based on the polylactic acid (PLA) filament, and is shown in Figure 12d. The acoustic experiments are executed and analyzed in an anechoic chamber that has been verified to exhibit partial conformance with ISO 3745 for free field and hemi-free field conditions [33]. The microphone (ECM8000, Behringer, Tortola, British Virgin Island), computer-connected audio device (Quad-Capture, Roland, Hamamatsu, Japan), and audio software (Sonar X2 Studio, Cakewalk, Boston, MA, USA) are identical to the experimental configuration of a previous paper [30] except for the sound generator. To replicate the near-field point source, a high-output in-ear monitor (UE900, Logitech, Lausanne, Switzerland) is mounted on the positioning device to control the distance and height of the sound source, as shown in the right-hand side of Figure 12d. The direction is observed by the line laser (GLL 3-80, Bosch, Stuttgart, Germany), and the distance is measured by a laser distance meter (DISTO D3a, Leica, Wetzlar, Germany). The system object by Matlab provides the real-time analysis and optimization for the structure via the direct connection to the audio devices. The results from the acoustic experiment in the environment described above are studied and organized in the next section.

## 4. Results

**Figure 13:**
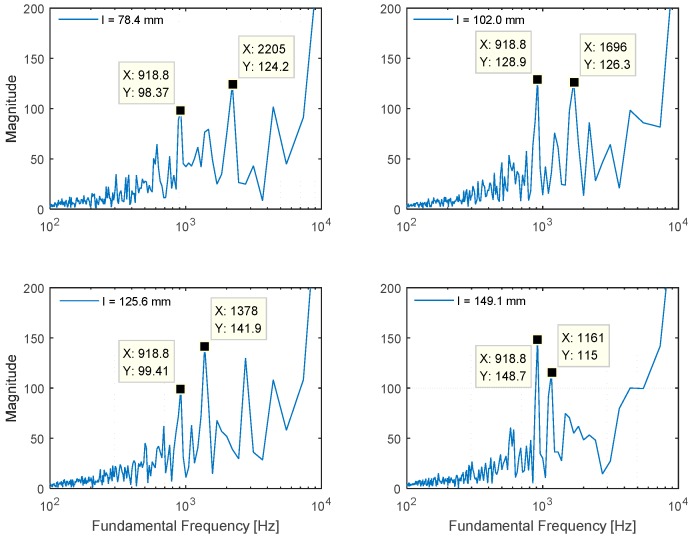
Plot matrix for fundamental frequency of NFML structure experiment with selected lengths.

The preliminary theory and extensive simulations in the previous section were used to develop the NFML structure with ten directional pipes. The selected lengths representing the particular directions are evaluated for a performance analysis in Figure 13. The data window length for the Cepstral parameter is 1024 samples, and 20 windows are ensemble averaged for a low variance spectrum outcome. Note that the sampling frequency is 44.1 kHz for all experiments. The 78.4-mm, 102.0-mm, 125.6-mm, and 149.1-mm-length pipes deliver 2205 Hz, 1696 Hz, 1378 Hz, and 1161 Hz fundamental frequencies, respectively, and the result is equivalent to the simulation counterpart in Figure 9. In addition, the fundamental frequency from the center body can be detected as 918.8 Hz in Figure 13. Within the limited range from *r_min_* to *r_max_* in Equation (4), the estimation process determines the local maximum in magnitude and derives the fundamental frequency for the received signal. The magnitudes from outside the range are not considered; hence, Figure 13 demonstrates suitable output for localization.

**Table 1:** Decision table between fundamental frequency and azimuthal direction.

*r* Index	9	10	11	12	13	14	15	16	17	18	19	20
**Corresponding Fund. Freq. (Hz)**	2450	2205	2005	1838	1696	1575	1470	1378	1297	1225	1161	1103
**Decision Length (mm)**		78.4	86.3	94.1	102.0	109.8	117.7	125.6	133.4	141.3	149.1	
**Decision Direction (°)**		336	300	264	228	192	156	120	84	48	12	
**Used Range in Gray**		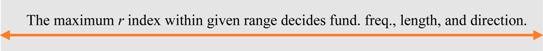										

According to the experiment, the local maximum location for individual length precisely represents the designed fundamental frequency based on simulations and equations. Table 1 shows the derived consecutive range for *r* of the Cepstral parameter and the corresponding fundamental frequency. Once the maximum magnitude *r* is evaluated from the Cepstral parameter based on the received signal, the value is sequentially translated to the fundamental frequency, length, and direction. Therefore, Table 1 serves as the direction model for estimation.

**Figure 14:**
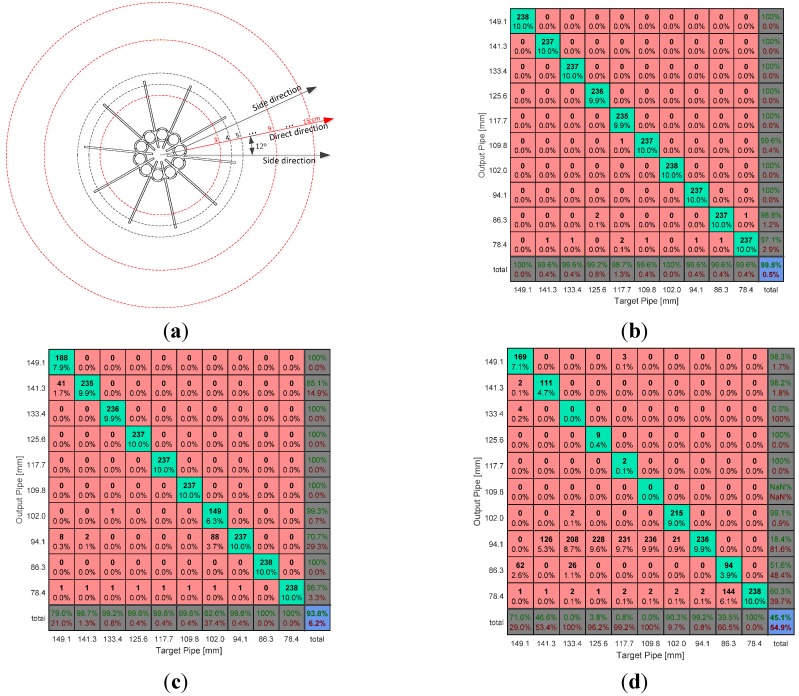
Experimental results: (**a**) Speaker locations for acoustic experiments. The azimuthal distance is measured from the outer body of the directional pipes, and the speaker height from the bottom is 96 mm; (**b**) confusion matrix for 3-cm distance (direct directions only); (**c**) confusion matrix for 9-cm distance (direct directions only); and (**d**) confusion matrix for 15-cm distance (direct directions only).

The data is recorded for 30 s in the assigned position with a 44.1 kHz sampling frequency. The first and last one second of data are removed to maintain the stationary characteristic of the signal. One dataset consists of 20 windows of 1024 samples in length for ensemble averaged estimation. Three-quarters of the dataset are overlapped with neighbor sets; therefore, the total number of datasets for the 28-second data is 238 (*i.e*., the number of iterations for performance evaluation). The azimuthal plane is divided into 30 directions (*i.e.*, 10 for the direct directions and 20 for the side directions). The direct direction is a linear path to each directional pipe inlet, and the side direction is ±12° off the direct path, as shown in Figure 14a. Note that the number of direct and side directions is proportional to the number of pipes in the NFML structure. The data is collected for all directions and for every centimeter distance from 3 to 15 cm. The distance is measured from the nearest outer body of the directional pipe.

Figure 14b–d show confusion matrixes for the 3-cm, 9-cm, and 15-cm distance results, respectively, for direct directions only with a constant sound source height at 96 mm from the bottom of the structure. The pipe length for each column vector of the matrix represents the target pipe length (condition) in which the signal is propagated. The length for each row vector stands for the output pipe (test outcome) that the NFML system determines. The green and red rates on the last row signify the true-positive rate (hit rate) and false-negative rate (miss rate) for each pipe length, respectively. In the bottom-right-hand corner, the overall values of the hit and miss rates are numerated. As the distance increases, the hit rate decreases substantially (*i.e*., from 99.5% to 93.8% to 45.1%). The 3-cm distance is the range within the wing perimeter of the NFML structure; therefore, the sound propagation is relatively converged on the line-of-sight (LoS) pipe inlet for a near-perfect hit rate. In contrast, the 15-cm result demonstrates that the lengths greater than or equal to 102.0 mm are decided to 94.1 mm in most cases. In other words, 1383 Hz is the dominant fundamental frequency for approaching far-field conditions due to acoustic diffraction.

**Figure 15:**
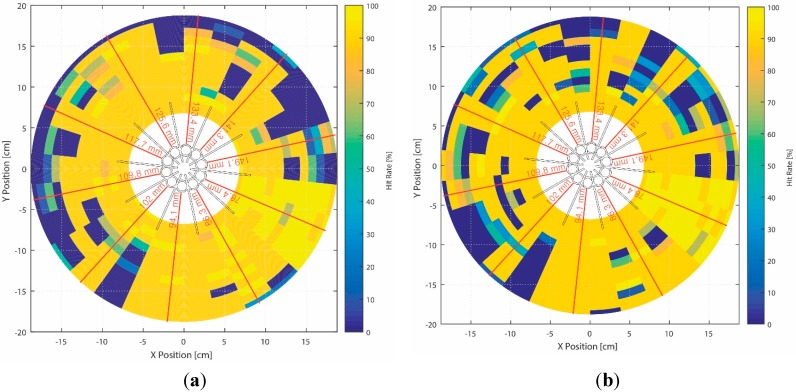
True-positive rate (hit rate) for all direct (red line) and side directions: (**a**) constant speaker height and (**b**) adjusted speaker height for inlet window level of each directional pipe.

Figure 15 presents the overall hit rate distribution for all directions and distances in a scaled illustration. The NFML structure is located at the center in black, and the red radial lines represent the direct directions for the corresponding pipe lengths. Figure 15a shows the constant height result for the sound source at 96 mm from the bottom of the structure, and Figure 15b shows the counterpart for the adjusted source to the level of the pipe inlet. Both figures show that the direct directions and immediate distances produce an increased hit rate in general. In particular, within the wing perimeter, high estimation accuracy is observed for any configuration. Comparing the outcomes of both sound height arrangements, the adjusted situation renders slightly worse performance than the constant situation. In particular, the side directions display perceptible performance variations; however, the global pattern of performance is almost equivalent. The hit rates of far-end distance approach collapsing the performance barrier in nearly all directions except short pipe lengths in the south and southeast directions due to acoustic diffraction. Longer distances are expected to produce less accurate estimations on average.

**Figure 16:**
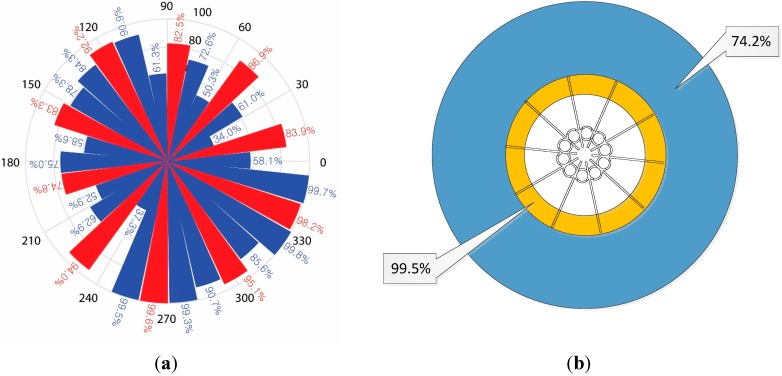
Average true-positive rate (hit rate) for (**a**) direct (red) and side (blue) directions and (**b**) inner and outer wing area.

Figure 16 visualizes the spatially averaged hit rate for the independent directions and areas. The directional results in Figure 16a represent the direct directions in red and the side directions in blue with the radial length proportional to the hit rate. The improved hit rate is produced by the direct directions and the shorter-length pipes. Notably, the side directions around the short-length pipes yield high accuracy as well. Figure 16b divides the circular area for the inside (up to 4 cm) and outside (from 5 cm) of the wing perimeter. The inside outperforms the outside in terms of hit rate with a significant difference due to the acoustic diffraction that is dominant for the farther distances between the sound source and NFML structure.

## 5. Conclusions

This paper presents a novel method of localizing the arrival direction of a near-field sound source with a single microphone. The asymmetric structure around the microphone produces direction-wise spectral variation, which can be identified by a dedicated algorithm to estimate the sound source position. The cylindrical pipes with designated lengths to generate the specific fundamental frequency are arranged vertically to maintain the small profile. The ten pipes with wave inlets and outlets receive and collect the acoustic signal to the microphone. The obtained signal is processed by the Cepstrum procedure to estimate the fundamental frequency that provides the arrival direction. Acoustic diffraction is caused by the relatively undersized structure; hence, the radial wings are utilized to increase the virtual volume for the near-field situation. The acoustic experiments in the anechoic chamber show that the direct and side directions deliver average hit rates of 89% and 73%, respectively. The closer positions to the system demonstrate higher accuracy, and the overall hit rate performance is 78% up to 15 cm away from the structure body.

The preceding work [30] on the ML system focused on a far-field sound source with a relatively bulky structure (*i.e*., 94 cm in maximum diameter and 5 cm in depth). The design procedure was implemented by the mathematical modeling of an individual substructure only; therefore, the optimization was performed with fewer degrees of freedom for far-field localization. The structure chose the origami-style realization due to the oversized scale. The purpose of this article is identical to that of the previous paper except the range of estimation here focuses on the near-field condition. Moreover, the dimensions of the structure are significantly reduced (*i.e*., 14 cm in wing-to-wing diameter, 5 cm in main body diameter, and 19 cm in depth). A 3D printer is employed for precise creation. The fine-grained simulation is included in the design procedure along with the preliminary modeling of the acoustic resonance; hence, the localization accuracy is improved considerably for the near-field sound source. Note that both works are realized by an identical algorithm for fundamental frequency estimation. Therefore, the low-complexity computation and hardware of the ML system are maintained with an improved structure in the NFML localization of this paper.

The enhanced structure and algorithm provide increased estimation performance for the ML system in not only near-field but also far-field situations. The potential for selection in terms of acoustic structures and estimation algorithms is considerable. Together with the continuous structure for high-resolution localization, future works will include the utilization of various structure tubes. Moreover, the resonance in the cavity, known as the Helmholtz resonance, will exhibit the appropriate choice for modifying the incident wave spectrum. This paper employs DFT-based signal processing to search for the numeric solution of the estimation. Future works will devise a mathematical model to represent the received frequency information by its coefficients as a parametric method. The similarity between the consecutive datasets will be explored by temporal post-processing in a statistical manner. With all of the above, the development of a 3D ML for the azimuthal and elevation directions is the final objective of this research.
